# Editorial: Thrombotic Microangiopathies, Diagnostic and Therapeutic Advances

**DOI:** 10.3389/fmed.2021.778352

**Published:** 2021-11-29

**Authors:** Robert W. Maitta, Jay S. Raval, Hollie M. Reeves, Magali J. Fontaine

**Affiliations:** ^1^Department of Pathology, Case Western Reserve University, Cleveland, OH, United States; ^2^Department of Pathology, University of New Mexico, Albuquerque, NM, United States; ^3^Department of Pathology, University of Maryland, Baltimore, MD, United States

**Keywords:** thrombotic microangiopathy, thrombotic thrombocytopenic purpura, diagnosis, therapy, microangiopathic hemolytic anemia, drug-induced, atypical hemolytic uremic syndrome

Thrombotic microangiopathies (TMAs) as a disease group represent a diagnostic and clinical challenge to practitioners. Presentations, which at times have overlapping symptomatology or have undetermined etiologies, emphasize the need for a constant reassessment of what is known about this disease group in order to determine not only how to best treat patients but also to develop targeted approaches to test for a given etiology. Likewise, in those instances when excluding diagnoses is the only available option, timely adjustment of therapy due to lack of therapeutic response needs to be achieved in the most efficient way. It is with this in mind that this topic-specific collection of articles was conceptualized to provide a forum to review not only what is currently known about these disease entities but also to present new findings in order to keep readers abreast of ways in which these diseases can be discerned. This is important since each of these etiologies responds to different therapies with a distinct road to recovery.

Sometimes diseases at presentation may have a preponderance in complement pathway activation characterized by C3 deposition similar to cases of immune-complex membranous glomerulonephritis (Hanna et al.). In these patients, thrombocytopenia may not be as significant as their renal impairment, and complement abnormalities are at the center of their presentation. This is discussed in more detail in this issue whereby using a case-driven approach, contributors suggest that genetic testing by itself may not always lead to the discovery of complement-related mutations. Instead, C3 staining patterns or presence of anti-factor H antibodies can ultimately lead to rapid recognition and initiation of appropriate therapeutic regimens while patients await renal transplantation. Along the same lines, complement mediators can not only mediate disease pathology but also serve as biomarkers in cases when thrombocytopenia occurs post-hematopoietic stem cell transplantation. In such patients, a report in this issue indicated that increases in soluble-C5b-9 can be seen and may be a diagnostic marker of patients who develop transplant-associated TMA (Mezö et al.).

Thrombocytopenia can also be an unintended adverse event from an increasing list of medications that include chemotherapeutic agents, monoclonal antibodies, and antimicrobials, to name a few. Unfortunately, this can go unrecognized in clinical practice and remain lower in the differential due to a lack of clinical suspicion. Adding complexity to this problem, mechanisms leading to drug-induced TMA are likely distinct from one another due to the different drug agents involved and intended corresponding cellular targets; however, this TMA may involve either antibody formation or dose-dependent and cumulative toxicity, and recovery occurs upon discontinuation of the offending drug (Chatzikonstantinou et al.). This is discussed in this issue where an update of drugs leading to this type of TMA and potential mechanisms are reported.

It is understandable that when clinicians see a patient with acute thrombocytopenia in the presence of schistocytes in the peripheral smear, a very non-specific test without standardization, the *gravitas* of the clinical picture may make them think of the most serious presentation. This is the case with thrombotic thrombocytopenic purpura (TTP), which is associated with high morbidity and mortality, requiring prompt initiation of therapy in the form of therapeutic plasma exchange (TPE) and concurrent immunosuppression. The latter specifically can be a matter of concern to practitioners because current literature is still not clear on which long-term immunosuppression regimen best leads to platelet count recovery and reduces relapses. It is thus encouraging that a report in this issue found similar efficacy in treating TTP patients with either rituximab or cyclophosphamide (Abou-Ismail et al.). Nowadays, it is well-accepted that the test to confirm the clinical diagnosis of TTP is ADAMTS13 activity. However, this test is often performed at reference laboratories and results may not be available at the time of therapy initiation. Notably, it should be of interest to readers that a broad platelet differential that focuses on immature platelets produced at the bone marrow can aid in differentiating TTP from other thrombocytopenias (Reeves and Maitta). On the other hand, in those thrombocytopenic cases in which ADAMTS13 activity is normal, a comprehensive body survey may be required to identify possible metastatic malignancies that have gone unnoticed (Osti et al.). With the advent of caplacizumab, the first TTP-specific approved therapy, it should be of interest that cases refractory to TPE and steroids respond to the agent even when initiated during the second week of hospitalization leading to recovery from thrombocytopenia and thrombi-related complications such as neurological impairments (Mellaza et al.). Finally, this issue will discuss congenital TTP unveiled by an infectious agent that led to a thorough workup that resulted in proper diagnosis and successful therapy (Wendt et al.).

In clinical practice, TMA patients can have a broad range of symptomatology and a report looking at almost 300 patients diagnosed with TMA serves as a good source to exemplify the challenges when encountering such patients (Henry et al.). Without a doubt, the vast majority of patients will have a TMA secondary to a primary insult rather than a primary TMA such as TTP or atypical hemolytic uremic syndrome (aHUS). This study stresses the need of proper testing that does not neglect ADAMTS13 activity and/or complement mutation studies, when applicable, to avoid misdiagnosing patients and yielding an accurate and timely diagnosis (Henry et al.). Notably, another contribution to this collection brings attention to and argues in favor of potentially new complement circulating factors mediating familial cases of aHUS when mutations in known complement genes are not identified (Piras et al.).

To conclude, as new discoveries in the field of TMA research that include reports of new highly-specific therapies in the form of monoclonals, among others, come to fruition, the future looks promising that some of these diseases will be treated in a targeted way earlier in the clinical course resulting in improved patient outcomes. Thus, it is the hope of the topic editors and contributors of this special collection that it will serve as a conduit to continue the discussion of TMA presentations while providing guidance on how to best treat patients under our care.

## Author Contributions

All authors contributed to writing and editing the manuscript.

## Conflict of Interest

The authors declare that the research was conducted in the absence of any commercial or financial relationships that could be construed as a potential conflict of interest.

## Publisher's Note

All claims expressed in this article are solely those of the authors and do not necessarily represent those of their affiliated organizations, or those of the publisher, the editors and the reviewers. Any product that may be evaluated in this article, or claim that may be made by its manufacturer, is not guaranteed or endorsed by the publisher.

